# The atlas of StW 573 and the late emergence of human-like head mobility and brain metabolism

**DOI:** 10.1038/s41598-020-60837-2

**Published:** 2020-03-16

**Authors:** Amélie Beaudet, Ronald J. Clarke, Jason L. Heaton, Travis R. Pickering, Kristian J. Carlson, Robin H. Crompton, Tea Jashashvili, Laurent Bruxelles, Kudakwashe Jakata, Lunga Bam, Luc Van Hoorebeke, Kathleen Kuman, Dominic Stratford

**Affiliations:** 1 School of Geography, Archaeology and Environmental Studies, University of the Witwatersrand, Private Bag 3, Johannesburg, WITS 2050 South Africa; 2 Department of Anatomy, University of Pretoria, PO Box 2034, Pretoria, 0001 South Africa; 3 Evolutionary Studies Institute, University of the Witwatersrand, Private Bag 3, Johannesburg, WITS 2050 South Africa; 4 Department of Biology, Birmingham-Southern College, 900 Arkadelphia Road, Birmingham, AL 35254 United States; 5 Plio-Pleistocene Palaeontology Section, Department of Vertebrates, Ditsong National Museum of Natural History (Transvaal Museum), 432 Paul Kruger Street, Pretoria Central, Pretoria, South Africa; 6 Department of Anthropology, University of Wisconsin, Madison, WI 53706 United States; 7 Department of Integrative Anatomical Sciences, Keck School of Medicine, University of Southern California, 1975 Zonal Avenue, Los Angeles, CA 90033 United States; 8 Department of Musculoskeletal Biology, Institute of Ageing and Chronic Disease, University of Liverpool, William Henry Duncan Building, W Derby Street, Liverpool, L7 8TX United Kingdom; 9 Molecular Imaging Center, Department of Radiology, Keck School of Medicine, University of Southern California, 2250 Alcazar Street, Los Angeles, CA, 90033 United States; 10 Department of Geology and Paleontology, Georgian National Museum, 3 Shota Rustaveli Ave, T’bilisi, 0105 Georgia; 11 French National Institute for Preventive Archaeological Researches (INRAP), 561 rue Etienne Lenoir, 30900 Nîmes, France; 12 French Institute of South Africa (IFAS), USR 3336 CNRS, 62 Juta Street, Braamfontein, Johannesburg, 2001 South Africa; 13 South African Nuclear Energy Corporation SOC Ltd. (Necsa), Elias Motsoaledi Street Ext. (Church Street West), R104 Pelindaba, North West Province South Africa; 14 UGCT Department of Physics and Astronomy, Ghent University, Proeftuinstraat 86/N12, B-9000 Gent, Belgium

**Keywords:** Anthropology, Palaeontology

## Abstract

Functional morphology of the atlas reflects multiple aspects of an organism’s biology. More specifically, its shape indicates patterns of head mobility, while the size of its vascular foramina reflects blood flow to the brain. Anatomy and function of the early hominin atlas, and thus, its evolutionary history, are poorly documented because of a paucity of fossilized material. Meticulous excavation, cleaning and high-resolution micro-CT scanning of the StW 573 (‘Little Foot’) skull has revealed the most complete early hominin atlas yet found, having been cemented by breccia in its displaced and flipped over position on the cranial base anterolateral to the foramen magnum. Description and landmark-free morphometric analyses of the StW 573 atlas, along with other less complete hominin atlases from Sterkfontein (StW 679) and Hadar (AL 333-83), confirm the presence of an arboreal component in the positional repertoire of *Australopithecus*. Finally, assessment of the cross-sectional areas of the transverse foramina of the atlas and the left carotid canal in StW 573 further suggests there may have been lower metabolic costs for cerebral tissues in this hominin than have been attributed to extant humans and may support the idea that blood perfusion of these tissues increased over the course of hominin evolution.

## Introduction

Because of its role in posture, locomotion and overall trunk stability and mobility, the vertebral column represents a key region for reconstructing mammal behaviour (e.g.,^1–3^). In particular, the atlas, or first cervical vertebra, acts as the main interface between the head and the axial skeleton and, therefore, plays a critical role in directing and stabilizing head movements (rev. in^4,5^). As such, previous studies have emphasized the functional link between the morphology of the atlas, head mobility, and postural and locomotor repertoires in catarrhines (e.g.^5–7^). More specifically, while the shape and curvature of the articular facets at the atlanto-occipital joint determines the range of head motions in terms of flexion and extension, the atlanto-axial joint is responsible for lateral rotation of the atlas and the head (rev. in^4–8^). Combined analysis of the superior and inferior articular facets has the potential to provide essential information about the range of head motions in *Australopithecus*, which has only been superficially assessed due to the scarcity and fragmentary nature of cervical vertebrae in the hominin fossil record^9,10^. Indeed, a non-human hominoid-like atlas in *Australopithecus* (e.g., concave superior and inferior articular facets) might suggest a range of head motions that differs from humans^6,7,11,12^, indicating that arboreal activities were fundamental components of the postural and locomotor repertoire of *Australopithecus*. Additionally, confirming the presence/absence of soft-tissue characters of the neck of *Australopithecus* (e.g., nuchal ligament^13,14^) would be crucial for our understanding of the evolutionary history of key specificities in human behaviour (e.g., endurance running^15^). Accordingly, the study of the atlas may offer new perspectives on the critical issue of whether or not adaptations for terrestrial bipedalism resulted in reduced ability in arboreal activities in *Australopithecus* and on the timing and mode of the emergence of human range of head motion (rev. in ^16,17^).

Critically evaluating hypotheses relating changes in hominin neurology (i.e., organization of the cortex, interneuron connectivity; as hypothesised by Holloway^18^, Holloway *et al*.^19^, Seymour *et al*.^20^) with changes in the diet and behaviour (i.e., a shift to a high-quality diet with stone-tool-assisted consumption of meat^21,22^), is exceptionally difficult when relying solely on the fossil record. In addition to serving as an essential structural component of the segmented axial skeleton connecting the cranium to the vertebral column (while allowing movement of the former relative to the latter), the atlas also functions as a conduit for arteries ascending through the cranium and supplying blood to the brain^23^. Recent studies have revealed that the cross-sectional area of the transverse foramina in the cervical spine could be used as a reliable proxy for estimating blood flow and brain metabolism in euarchontans (including hominoids), especially when combined with an assessment of dimensions of the carotid foramina^24^. Given the paucity of studies evaluating brain metabolism in *Australopithecus*, even an indirect measure of brain perfusion is important to investigate^20,21,25,26^. Assessments of total encephalic arterial flow (i.e., in arteries running through the cervical vertebrae and the carotid canal) could help in estimating blood flow and potential variation in brain energetic demands characterizing early hominins, and could confirm (or not) a relatively recent emergence of the human-like metabolic pattern in the hominin lineage (i.e., having a brain that accounts for a relatively high percentage of the estimated basal metabolic rate)^20,24^.

The relative scarcity of the first cervical vertebra in the fossil record of *Australopithecus* and other hominins and the fragmentary nature of the few specimens that have been discovered thus far mean that extensive speculation is needed when discussing the form of the atlas in human evolution. To date, only four fragmentary atlases attributed to *Australopithecus* have been described, i.e., the AL 333-83 atlas from Hadar^27^, the atlas of the *Australopithecus afarensis* infant DIK-1-1 from Dikika^28^ and fragments of two first cervical vertebrae attributed to *Australopithecus anamensis* from Assa Issie^10^. Here we significantly advance this knowledge base by describing the atlas from StW 573 (‘Little Foot’), a 3.67 million-year-old *Australopithecus prometheus* specimen from Sterkfontein Member 2 comprised of a skull and associated postcranial skeleton^29–34^. This specimen represents the most complete early hominin adult first cervical vertebra in the fossil record. In addition, we describe a newly discovered partial atlas from Sterkfontein Member 4 (StW 679), the first hominin atlas ever found in this stratigraphic unit (location U/43, 6′10′′–7′10′′). Through comparative study of these two fossils, we quantitatively investigate the shape of the hominin atlas, with a particular emphasis on the articular facets, and comment on the selective pressures that may have been operating on head mobility in *Australopithecus*. Given the exceptional degree of preservation of StW 573, we also evaluate the relative blood supply of the vertebral arteries and internal carotid arteries contributing to brain metabolism in *Australopithecus* and discuss related selective pressures.

## Results

### Description and surface-based comparisons

**Figure 1:**
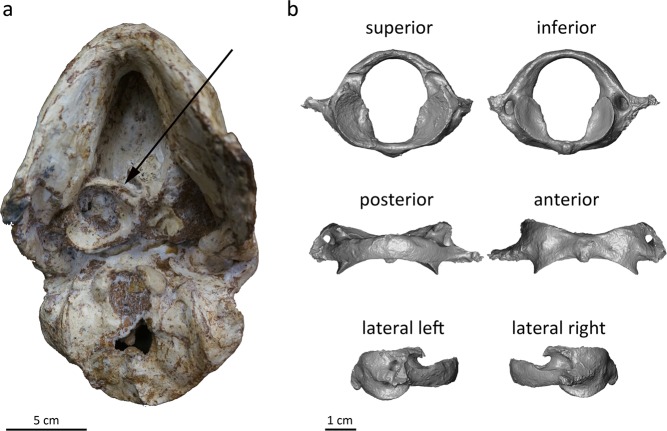
The StW 573 atlas. (**a)** Photograph of the StW 573 atlas cemented by breccia to the cranial base anterior to the foramen magnum. (**b)** In silico digital reconstruction of the atlas of StW 573 based on microtomographic data.

**Figure 2:**
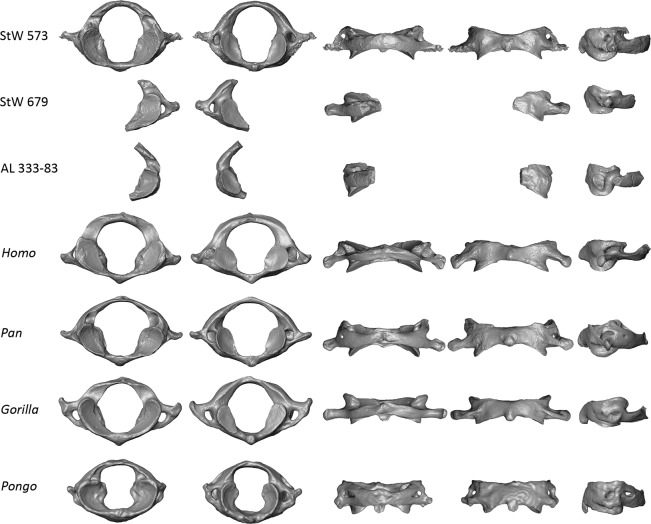
Comparison of the StW 573 atlas with extant and fossil specimens. Virtual renderings of the StW 573 atlas compared to the *Australopithecus* specimens StW 679 and AL 333-83 and to extant *Homo*, *Pan*, *Gorilla* and *Pongo*. Atlases are shown from left to right in superior, inferior, posterior, anterior and lateral left views, respectively. Images not to scale.

Following removal of the StW 573 skull from the Silberberg Grotto of the Sterkfontein Caves, further physical cleaning and micro-CT scanning revealed a nearly complete atlas cemented by breccia in its displaced and flipped over position (revealing the superior aspect) on the cranial base anterolateral to the foramen magnum (Fig. 1a; and see Figure 9 in Clarke and Kuman^30^). The atlas of StW 573 is missing only the left transverse process and exhibits slight damage to the tip of the right transverse process (Fig. 1b). StW 679 is a fragmentary atlas preserving the left inferior and superior articular facets as well as parts of the left transverse process, including the transverse foramen (Fig. 2). Neither specimen exhibits modification through plastic deformation.

**Figure 3:**
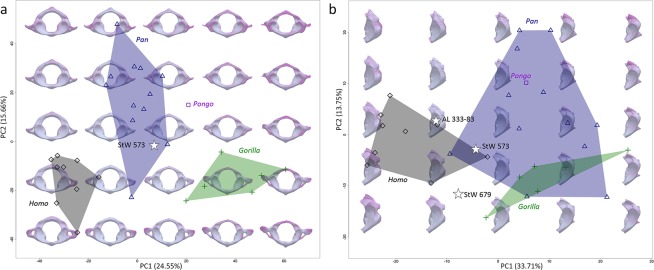
Statistical analyses of the StW 573 atlas and comparative extant and fossil specimens. Principal component analyses and backtransform morphospace of the deformation-based shape comparisons of the complete and partial atlases of StW 573, StW 679 and AL 333-83 and of extant *Homo*, *Pan*, *Gorilla* and *Pongo*. (**a)** Analysis of the complete atlases. (**b)** Analysis of the partial atlases.

**Figure 4:**
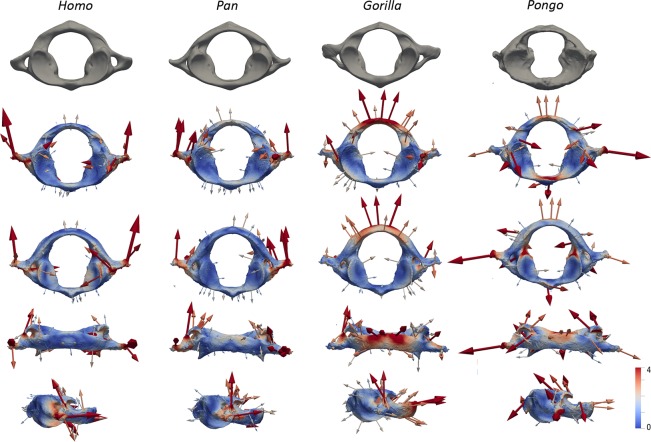
Surface-based comparison of the complete atlases of StW 573 and comparative extant specimens. Topographical distribution of the deformation-based shape comparisons of the complete atlases of StW 573 and of extant *Homo*, *Pan*, *Gorilla* and *Pongo*. Cumulative displacement variations from the taxon mean shapes to StW 573 are rendered by a pseudo-colour scale ranging from dark blue (lowest values) to red (highest values) visualised on the fossil individual surfaces. Vectors represent both the magnitude and orientation of the deformations needed to deform the taxon mean shapes to StW 573. Maximum value of the colour bar is considered to be the most appropriate compromise representation of both global and local deformations. Atlases are shown from top to bottom rows in superior, inferior, posterior and lateral right views, respectively.

**Figure 5:**
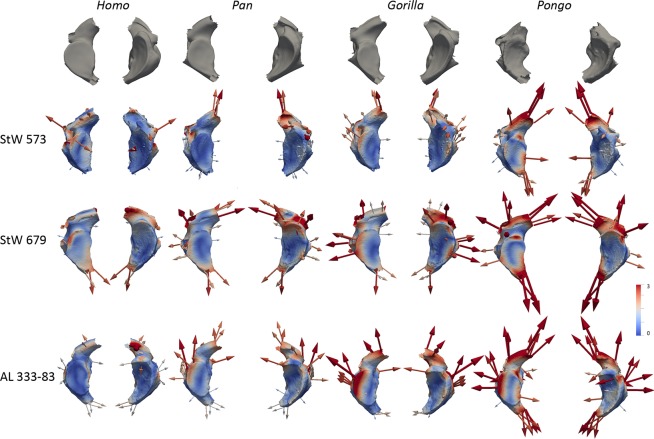
Surface-based comparison of fossil and extant partial atlases. Topographical distribution of the deformation-based shape comparisons of the partial atlases of StW 573, StW 679 and AL 333-83 and of extant *Homo*, *Pan*, *Gorilla* and *Pongo*. Cumulative displacement variations from the taxon mean shapes to the fossil specimens are rendered by a pseudo-colour scale ranging from dark blue (lowest values) to red (highest values) visualised on the fossil individual surfaces. Vectors represent both the magnitude and orientation of the deformations needed to deform the taxon mean shapes to StW 573. The maximum value of the colour bar is considered to be the most appropriate compromise representation of both global and local deformations. Atlas specimens within each of the four columns are shown from top to bottom rows in superior (left) and inferior (right) views, respectively.

In Fig. 2, morphologies of the StW 573 and StW 679 atlases are qualitatively compared to morphology of the *Australopithecus* atlas from Hadar AL 333-83^27^, as well as to those of extant specimens of *Homo* (i.e., throughout the manuscript we refer to *Homo* as extant representatives of *Homo* unless specified otherwise), *Pan*, *Gorilla* and *Pongo* (Supplementary Table S1). In addition to qualitative comparisons, we perform quantitative surface-based comparisons using a landmark-free registration method (see Methods). Results are statistically analysed by principal component analysis (PCA; Fig. 3). In addition, the mean shape of each extant comparative group is deformed to the StW 573 atlas, as well as to the StW 679 and AL 333-83 partial atlases, and colour maps and vectors are used to visualize resulting patterns of differences in magnitudes and orientations (Figs. 4–5). Moreover, both StW 679 and AL 333-83 are deformed to StW 573 (Supplementary Fig. S1). Because StW 679 and AL 333-83 are incomplete, we perform two separate analyses. One analysis includes the complete StW 573 atlas and extant comparative specimens, and a second analysis focuses exclusively on the left articular facets of the three fossil specimens and extant comparative specimens (see Methods; protocol published in Dumoncel *et al*.^35^ and Beaudet *et al*.^36^). Additionally, we compute a backtransform morphospace (i.e., representative shapes in a grid pattern across the PCA) that shows intuitively how shape varies across morphospace (Fig. 3).

Overall, morphology of the StW 573 atlas is closer to that of *Pan* than to any other atlas of extant comparative groups in the shape space of the PCA (Fig. 3a). When all of the fossil specimens are considered in the PCA analysis focusing on the left articular facets, StW 573, StW 679 and AL 333-83 plot along the first axis between the *Homo* cluster in negative space and the *Pan*, *Gorilla* and *Pongo* clusters in positive space (Fig. 3b). Morphologically, the superior articular facets in StW 573, StW 679 and AL 333-83 are intermediate between the non-human and human comparative taxa, being less steeply inclined than those of *Pan*, *Gorilla* and *Pongo* but more steeply inclined than those of *Homo* (Figs. 2–5). Additionally, the superior and inferior articular facets in StW 573 and AL 333-83 are more vertically oriented than in StW 679 and *Homo* and more closely approximate the condition seen in *Pan*, *Gorilla* and *Pongo* (Figs. 2–5). The vertebral foramen in StW 573 is human-like in being more expanded posteriorly (i.e., relative to the position of the inferior articular facets) than in any of the extant great apes (Figs. 2–4). The anterior tubercle is more developed in StW 573 than in *Homo* but, is less prominent than in *Gorilla* and *Pongo*, and most closely approximates the condition in *Pan* (Figs. 2 and 4). The transverse processes in StW 573 and StW 679 are more posteriorly oriented than in *Homo* relative to the position of the articular facets, and they are positioned similarly to those in *Pan*, *Gorilla* and *Pongo* (Figs. 2–4). Moreover, the transverse processes appear comparatively longer than those in *Homo* and *Pongo*, and approximate the relative length of those in *Pan* and *Gorilla*. The transverse foramina of StW 573 are situated inferior to the superior articular facets, as in *Pan* and *Pongo*, and unlike the more laterally positioned facets in *Homo* and *Gorilla* (Figs. 2–4). The retro-glenoid tubercles project cranio-dorsally in StW 573, unlike the retro-glenoid tubercle of StW 679 (this region is not well-preserved in AL 333-83; Figs. 2–5 and Supplementary Fig. S1). In posterior view, the posterior arch in StW 573 is relatively large supero-inferiorly and particularly smooth as compared to the non-human hominoid specimens with no prominent posterior tubercle (Figs. 2 and 4). Both StW 573 and StW 679 lack prominent tubercles for attachment of the transverse ligament, while damage to AL 333-83 prevents comparison (Fig. 2). In this respect the condition exhibited by StW 573 and StW 679 is similar to that of non-human hominoids. There are no *ponticulus posticus* in StW 573 (Fig. 1), or in extant *Homo*, while they are present in *Pan* and *Pongo* (Fig. 2). However, a *ponticulus lateralis* is present on the left transverse process in StW 573 (and absent/missing from the right side), and in *Pongo* on both sides (Fig. 2).

### Dimensions

Dimensions of the atlas of StW 573 are reported in Table 1 and compared with those of *Homo*, *Pan*, *Gorilla* and *Pongo* (see Methods; Supplementary Fig. S2). Most dimensions of the StW 573 atlas fall within the ranges of those expressed by *Pan* (i.e., AATh, MDvD, MTrD, M 11, PaTh, STrD, 1L, 1R), and *Pongo* to a lesser extent (i.e., MTrD, M 11, STrD, 2L), and are smaller or at the lower end of the *Homo* range. In contrast, the left and right articular facets (2R and 2L) are relatively large (i.e., in the range of *Homo* or larger than any of the comparative measurements). The estimated diameter along the major axis (1L) of the left articular facets of StW 679 is smaller than in any of the comparative groups except for *Pan*, while 2L falls within the ranges of those exhibited by *Homo*, *Pan* and *Gorilla*. The areas of the articular facets in StW 573 are within the ranges of all comparative samples with the exception of *Pan*, while the area of the left facet in StW 679 only fits *Pan*’s estimates. Anatomical ratios for the length and the width of the left articular facet in StW 573 and StW 679 fall within or close to the lower end of the *Homo* range, while the ratio of the right facet in StW 573 is more comfortably within the range of *Pan* despite a certain degree of overlap between the two groups. Dimensions of the articular facets in StW 679 are smaller than those in StW 573.

**Table 1:** Atlas osteological dimensions (in mm and mm^2^) of StW 573 and comparative material.

specimen/sample	AATh	MDvD	MTrD	M10	M11	PaTh	STrD	1L	2L	1L*2L	1L/2L	1R	2R	1R*2R	1R/2R
StW 573	4.5	35.5	61.5^†^	27.9	22.6	2.9	40.6	20.3	11.7	237.5	1.7	19.4	12.2	236.7	1.6
StW 679	**—**	**—**	**—**	**—**	**—**	**—**	**—**	15.7^‡^	9.8	153.9	1.6	**—**	**—**		
**extant** ***Homo*** **(*n*** = **9)**															
mean	5.9	42.6	72.3	30.2	27.5	6.8	48.3	22.5	11.0	248.5	2.1	22.1	9.8	216.7	2.3
range	4.4–8.1	38.2–44.5	64.6–78.4	27.4–32.2	26.1–30.0	3.4–8.5	40.6–52.7	19.4–26.3	8.5–13.4	165.9–320.2	1.7–2.6	20.1–24.9	8.3–11.7	189.4–251.6	1.9–2.9
s.d.	1.0	1.8	3.9	1.5	1.4	1.6	3.3	2.4	1.5	46.8	0.3	1.4	1.1	22.9	0.3
**extant** ***Pan*** **(*n*** = **12)**															
mean	4.7	33.9	60.3	24.7	23.2	4.1	43.8	17.8	7.5	133.9	2.4	18.3	7.7	140.6	2.4
range	3.2–6.9	27.9–39.7	45.2–69.5	22.0–27.6	21.2–25.0	2.1–7.0	36.5–49.6	14.7–20.7	6.4–10.1	101.1–204.6	2.0–3.0	14.1–26.6	6.9–11.5	97.7–208.0	1.6–3.7
s.d.	1.2	3.7	7.0	1.5	1.4	1.4	4.0	2.3	1.2	34.1	0.3	3.3	1.3	33.3	0.6
**extant** ***Gorilla*** **(*n*** = **6)**															
mean	8.1	46.9	85.4	34.4	31.7	4.3	54.6	26.0	10.1	264.7	2.6	25.5	10.0	255.5	2.6
range	5.7–10.2	40.0–56.6	71.5–96.2	29.4–37.9	27.3–35.8	2.2–11.1	48.3–58.6	22.4–28.4	8.9–13.1	211.1–367.5	2.2–3.0	22.8–29.5	8.0–11.4	182.0–307.0	2.2–2.9
s.d.	1.7	5.4	10.5	3.3	3.0	3.4	4.4	2.5	1.6	58.2	0.4	2.3	1.2	44.6	0.3
**extant** ***Pongo*** **(*n*** = **3)**															
mean	6.7	45.9	67.6	32.2	20.4	7.2	46.9	24.2	11.0	267.6	2.2	23.8	11.3	270.9	2.1
range	5.7–7.5	38.2–53.1	61.4–73.8	28.8–37.4	16.8–22.9	3.1–10.4	37.4–53.7	23.2–25.7	9.9–11.7	254.3–284.8	2.0–2.6	21.3–27.3	10.1–11.9	215.6–325.8	1.9–2.3
s.d.	0.9	7.5	39.5	4.5	3.1	3.7	8.5	1.3	1.0	15.6	0.3	3.1	1.0	55.1	0.2

AATh: anterior arch thickness; MDvD: maximum dorsoventral transverse diameter; MTrD: maximum transverse diameter; M10: canal dorsoventral maximum diameter; M11: canal transverse maximum diameter; PaTh: posterior arch thickness; s.d.: standard deviation; STrD: superior transverse diameter; 1L: diameter in major axis of the superior left articular facet; 2L: diameter at a right angle to 1L of the superior left articular facet; 1R: diameter in major axis of the superior right articular facet; 2R: diameter at a right angle to 1R of the superior right articular facet; 1L*2L and 1R*2R: estimation of the area of the articular surface (mm^2^) through the product of the diameter in major axis (1L/R) and the orthogonal diameter (2L/R) of each facet; 1L/2L and 1R/2R: ratio between the diameter in the major axis and the orthogonal diameter (see Supplementary Fig. S2).

^†^Estimate based on reconstruction of the left transverse process (see Methods).

^‡^Since the most anterior part of the left articular facet is not preserved, here we provide a minimum estimate only.

### Vertebral foramen and transverse foramina

We measure areas of the vertebral foramen and foramina of the transverse processes in StW 573 and comparative specimens (Table 2; Supplementary Table S1 and Fig. S2). Absolute areas of the StW 573 vertebral foramen and right and left transverse foramina fall within the range of those for *Pan*. Within the limits of our sample, standardized areas of the vertebral foramen and transverse foramina (i.e., areas divided by the product of length and width of the atlas; see Methods) in StW 573 fall within the ranges of all extant comparative groups. The absolute area of the left transverse foramen in StW 679 is smaller than those of *Homo*, *Gorilla* and *Pongo,* but is similar to those of *Pan* and StW 573.

**Table 2:** Absolute (in mm^2^) and scaled (dimensionless) cross-sectional areas of the vertebral foramen and the transverse foramina in StW 573 and comparative material.

specimen/sample	VFA	RFA	LFA
StW 573	474.9	17.5	17.6
	(21.7)	(0.8)	(0.8)
StW 679	—	—	16.3
**extant** ***Homo*** **(n = 9)**			
mean	604.7	35.9	36.7
	(19.7)	(1.2)	(1.2)
range	525.4–697.2	23.4–69.1	25.4–60.6
	(17.2–22.5)	(0.8–2.2)	(0.8–1.9)
s.d.	57.4	13.6	11.1
	(1.9)	(0.4)	(0.3)
**extant** ***Pan*** **(n = 12)**			
mean	417.6	21.1	20.2
	(21.0)	(1.1)	(1.0)
range	360.9–483.5	11.3–31.1	8.8–29.4
	(15.9–28.5)	(0.5–1.8)	(0.4–1.8)
s.d.	30.6	5.6	6.0
	(4.1)	(0.4)	(0.4)
**extant** ***Gorilla*** **(n = 6)**			
mean	868.7	37.7	35.3
	(21.7)	(0.9)	(0.9)
range	631.0–1073.0	20.5–57.4	24.2–46.4
	(19.2–24.7)	(0.6–1.1)	(0.7–1.0)
s.d.	163.1	13.7	9.0
	(2.0)	(0.2)	(0.1)
**extant** ***Pongo*** **(n = 3)**			
mean	541.6	23.0	24.3
	(19.1)	(0.8)	(0.9)
range	498.5–575.9	19.7–26.3	21.2–27.4
	(14.7–23.5)	(0.5–1.1)	(0.5–1.2)
s.d.	39.5	13.7	14.3
	(11.9)	(0.6)	(0.6)

VFA: area of the vertebral foramen; RFA; area of the right transverse foramen; LFA: area of the left transverse foramen; s.d.: standard deviation.

Mean, range and standard deviation values without parentheses are unscaled (in mm^2^), whereas values within parentheses are scaled (dimensionless). Values are scaled by dividing areas by the product of length and width of the atlas (see Methods). There is no scaled measurement for StW 679 due to incomplete preservation of the atlas.

###  Carotid canal

As comparative samples, we consider extant specimens of humans and chimpanzees as well as southern African specimens of *Australopithecus*, *Paranthropus* and early *Homo* (see Methods and Supplementary Table S2). To estimate brain perfusion, we measure cross-sectional areas of the carotid canal in the basicranium of StW 573 (left side) and the comparative material (Table 3; Supplementary Table S2 and Fig. S3). Absolute cross-sectional area of the StW 573 left carotid canal falls within the reported range for *Australopithecus* and is close to *Paranthropus*. Cross-sectional area of the carotid canal in StW 573, like that of the other fossil hominins included in this study, falls within the range of variation of *Pan*, but below the range exhibited by extant *Homo* (with no overlap between the ranges of variation of the two groups, Table 3).

**Table 3:** Cross-sectional area of the carotid canal (in mm^2^) in StW 573 and comparative material.

specimen/sample	side	CSA
StW 573	L	11.1
***Australopithecus***		
Sts 5	L	9.3
Sts 19	L	11.8
Sts 25	L	10.9
StW 53	L	19.0
StW 98	R	11.4
StW 329	R	8.1
StW 498	L	12.7
MLD 37/38	R	7.1
mean		11.3
range		7.1–19.0
s.d.		3.6
***Paranthropus***		
SK 47	L	10.7
**early** ***Homo***		
SK 847	L	14.0^†^
**extant** ***Homo*** **(n = 10)**	L	
mean		29.6
range		23.8–38.3
s.d.		5.0
**extant** ***Pan*** **(n = 5)**	L	
mean		13.6
range		6.9–20.8
s.d.		5.2

CSA: cross-sectional area; L: left; R: right; s.d.: standard deviation.

^†^Measured on non-homologous plane (see Methods).

### Brain glucose utilization

We estimate brain glucose utilization (BGU) in StW 573 by using the equation provided by Boyer and Harrington^24^ (see Methods; Supplementary Table S3). Brain cost in StW 573 is about three times lower than in extant *Homo*, and it fits more closely within the range observed in extant *Pan*. Using basal metabolic rates (BMR) provided by Boyer and Harrington^24^ for *Homo* (1557 kcal/day) and *Pan* (1370 kcal/day), we computed that StW 573 would have used 6.0% or 7.5% respectively of its BMR to support the brain, while *Homo* and *Pan* actually use 27.0% and 9.7% of their respective BMR. Even if the lack of direct association between crania and axial skeletons prevents firm conclusions for the other *Australopithecus* specimens included in our study, our estimations for specimens from Sterkfontein Member 4 and Makapansgat Member 4 concur with the observations for StW 573 and suggest lower brain cost compared with extant humans. When considering the range of variation estimated for StW 573 (Table 4), the minimum and maximum values for BGU are below the range of *Homo* and more closely approximate the range of *Pan* as reported in Table S3.

**Table 4:** Estimation of the range of brain glucose utilization (BGU) in the fossil specimens.

specimen/sample	− 1 s.d. *Homo*		+ 1 s.d. *Homo*		− 1 s.d. *Pan*		+ 1 s.d. *Pan*	
	ACA	BGU	ACA	BGU	ACA	BGU	ACA	BGU
StW 573	32.6	53.3	82.0	156.9	45.6	79.0	69.0	128.2
***Australopithecus***								
Sts 5	29.0	46.5	73.4	137.9	39.0	65.8	63.4	116.1
Sts 19	34.0	56.0	78.4	148.9	44.0	75.8	68.4	126.9
Sts 25	32.2	52.6	76.6	144.9	42.2	72.1	66.6	123.0
StW 53	48.4	84.7	92.8	181.4	58.4	105.5	82.8	158.7
StW 98	33.2	54.5	77.6	147.1	43.2	74.1	67.6	125.2
StW 329	26.6	42.0	71.0	132.6	36.6	61.1	61.0	111.0
StW 498	35.8	59.5	80.2	152.9	45.8	79.4	70.2	130.9
MLD 37/38	24.6	38.4	69.0	128.2	34.6	57.2	59.0	106.8
mean	33.0	54.3	77.4	146.7	43.0	73.9	67.4	124.8

ACA: total arterial cross-sectional area (mm^2^), BGU: brain glucose utilization; s.d.: standard deviation.

For each *Australopithecus* individual besides StW 573, we use the estimate of cross-sectional area of the transverse foramen of StW 679 for a proxy value and combine these with cross-sectional areas of carotid canal measures provided in Table 3.

## Discussion

Our study of the nearly intact StW 573 atlas and of the partial specimen StW 679 offers a unique opportunity to provide new insights into kinematics of head-neck movements and the neurovascular system in *Australopithecus*. The overall dimensions, shape of the articular facets, position of the transverse processes, and degree of development of the anterior tubercle in StW 573 are similar to *Pan*, while shape of the vertebral foramen and position of the posterior arch are more similar to *Homo*. When compared to the two other available fossil hominins, StW 573 shares vertically oriented articular facets with AL 333-83, while orientation of the facets in StW 679 is more similar to *Homo*. Intriguingly, the posterior arch in StW 573 is larger supero-inferiorly and smoother as compared to the extant hominoids. Moreover, both StW 573 and StW 679 lack prominent tubercles for attachment of the transverse ligament. Again, the area of the vertebral foramen and transverse foramina in both StW 573 and StW 679, and the area of the carotid canal in StW 573, fall within ranges for *Pan* and are smaller than *Homo*. Overall, our study suggests substantial similarities in shape and size of StW 573 and StW 679, with those of extant non-human hominoids, particularly *Pan*.

The musculoskeletal system of the neck is of prime interest for reconstructing kinematics of head-neck movements. Functional signals from the atlas derive from two main sources of evidence, i.e., the articular facets and the muscle and ligament insertions. The superior facets are in contact with the occipital condyles and are responsible for facilitating neck inclination angles and head movement repertoires, specifically flexion-extension in the sagittal plane^6–8,11,12^. Together with the presence of cranio-dorsally projecting retro-glenoid tubercles^6,11^, greater surface curvature in the atlanto-occipital articulations has been suggested to increase joint angular excursion in the sagittal plane, thus contributing to a higher range of sagittal motion (rev. in^7^). An increased range of sagittal motion might confer advantages for arboreal species because of the greater necessity of maintaining the visual field towards substrates arranged more generally in three dimensions^37^. More specifically, vertical climbing and suspensory activities both implicate visual inspection for substrates above and below^38^. Moreover, since the atlanto-axial joint plays a role in the axial rotatory movements (rev. in^8^), the shape of the inferior articular facets might constrain movements in the transversal plane.

In concert with prominent retro-glenoid tubercles, more concave superior articular facets and more vertically oriented inferior articular facets in StW 573 (i.e., non-human hominoid-like) may contribute to an adaptive complex of features that suggests a kinematic signal corroborating arboreal behaviour. Such a complex would be consistent with a study of the vestibular apparatus of StW 573 that demonstrates that the vertical canals, which provide feedback on head movements in sagittal and coronal planes^39^, are more similar to those of extant *Pan* than to those of *Homo*^40^. Finally, modern human-like orientation of the superior and inferior articular facets and the absence of prominent retro-glenoid tubercles in StW 679 might reflect greater selection for arboreal activities in earlier *Australopithecus* compared to this later Sterkfontein Member 4 *Australopithecus* specimen. This is highlighted by the more non-human, ape-like configuration exhibited in both StW 573 from Sterkfontein Member 2 and AL 333-83 from Hadar even if the identification of derived features in the 4.2 million-years-old partial atlases of *A. anamensis* from Assa Issie raises critical questions on evolutionary polarity of such traits^10^. Alternatively, the features of StW 679 may reflect the fact that it belongs to a different species, i.e., *A. africanus*^30^, although a larger comparative sample would be needed to interpret inter-individual differences.

The posterior vertebral arches of the cervical vertebrae, including the atlas, are connected to the head via nuchal muscles and the nuchal ligament. In StW 573, there are no enlarged areas of insertion for the attachment of muscles and ligaments on the posterior arch, and the surface is relatively smooth as compared to extant hominoids. This observation might support the hypothesis of the absence or reduction of the nuchal ligament in *Australopithecus*^13–15^. The relatively long length and dorsal orientation of the transverse processes in the StW 573 atlas indicate mechanical advantages similarly seen in the suboccipital muscles of *Pan* and *Gorilla* and in particular, in the muscles that move the pectoral girdle (i.e., atlanto-clavicularis, levator scapulae^4,7,14^). Such features might be interpreted as conferring potential selective advantages for arboreal activities^4,41^. The analysis of the scapula and clavicle of StW 573 will add further insight into functional capacity of the pectoral girdle^42^. Because of the role of the long muscles of the neck in controlling and maintaining neck posture and in counteracting the lordosis increment related to the weight of the head, relatively minimal development of the anterior tubercle in StW 573 might indicate a low degree of cervical lordosis as compared to extant humans and later *Australopithecus*^9,43^. Finally, StW 573 and StW 679 do not present prominent tubercles for attachment of the transverse ligament, which is unlike the condition seen in extant *Homo*. Gómez-Olivencia *et al*.^44^ suggested that ‘small tubercles’ might represent the derived condition in Neanderthal, while the prominent tubercles in *Homo sapiens* illustrate the primitive condition for *Homo*. However, the relatively high prevalence of small tubercles in *Australopithecus* might contradict this interpretation.

The area of the vertebral foramen in StW 573 is absolutely and relatively similar in size to *Pan*. The vertebral foramen notably accommodates the spinal cord and other neurovascular structures, including the internal vertebral venous plexus^23^. According to Falk’s hypothesis^45^, the shift to orthograde posture in hominin evolution would be correlated with changes in the vascular system resulting in blood draining preferentially to the vertebral plexus instead of into the internal jugular veins. Following this assumption, we might therefore expect changes in the size of the vertebral plexus in bipedal early hominins as compared to the ancestral pattern. However, StW 573 exhibits neither an absolutely nor relatively larger vertebral foramen than the comparative extant hominoids (nor extant *Homo* if we consider the relative values in Table 2, but see Meyer and Haeusler^46^ for lumbar regions). Nevertheless, shape of the vertebral foramen in the atlas does differ between StW 573 and extant *Homo* when compared with non-human hominoids, and an antero-posterior elongation and lateral compression of the canal in StW 573 and *Homo* could be consistent with a rearrangement of neurovascular structures passing through the vertebral canal. However, the rest of the StW 573 vertebral column must be investigated to assess vertebral foramen shape throughout the column and explore potential functional implications.

Variation in the size of transverse foramina and the carotid canal may provide evidence for estimating blood flow to the brain, informing about brain metabolism^24^. Our study reveals relatively smaller cross-sectional areas of transverse foramina and the carotid canal in StW 573 compared with extant *Homo*. Given that the vertebral arteries are bigger in species with bigger brains and the cranial capacity in StW 573 is similar to the extant chimpanzee values^24,47^, these results may be expected. It is interesting to consider that brain perfusion in extant great apes is suggested to be higher than in *Australopithecus*^48^. Thus, future analyses would have to investigate selective pressures that could explain the increase of brain perfusion over the last three million years of hominin evolution and if similar evolution could be detected in other primate lineages. In addition, our measurements of the total encephalic arterial flow (i.e., vertebral arteries and internal carotid arteries) and calculation of the brain glucose utilization (BGU) in StW 573 support a low brain metabolism in *Australopithecus* compared to that in *Homo*. This could suggest a relatively recent emergence of the human-like metabolic pattern in the hominin lineage^20^, if this pattern in StW 573 is representative of *Australopithecus*. Low investment in brain metabolism could be tentatively explained by a low quality diet in Pliocene *Australopithecus*, and more specifically to a low proportion of high-quality animal-based products^21,25^; but see Sponheimer and Thorpe^49^). Alternatively, if we consider the hypothesis of less efficient bipedalism in *Australopithecus* as compared to extant *Homo* (e.g.,^50,51^), high metabolic energy may have been required by upright walking gaits if these were of an obligate nature in this fossil taxon (but see previous simulations suggesting human-like bipedal performance in *Australopithecus*^52–54^). By extension, another possible explanation for the lower brain metabolism in StW 573 could be a higher investment in postural and locomotor activities that may reduce proportions of the metabolic rate that would have been available for allocation to the brain. The fact that body proportions may have not been adapted to maintain appropriate thermoregulation while walking bipedally in *Australopithecus* might support the latter scenario^55,56^.

## Methods

### Materials

As comparative materials, we included a cast of the specimen AL 333-83 from the Hadar Formation recovered during the field seasons of 1974–1977^27^ and that is currently housed at the Cleveland Museum of Natural History. AL 333-83 represents a partial atlas that preserves most of the left side, including the inferior and superior articular facets and a portion of the posterior arch, but it is missing the left transverse process^27^ (Fig. 2). Additionally, for measuring dimensions of the carotid canal, we investigated 10 southern African fossil hominin crania from the sites of Makapansgat (Member 4), Sterkfontein (Member 4) and Swartkrans (Member 1) (Supplementary Tables S1, S2; for further details see Beaudet *et al*.^40,47,57^).

Our comparative sample of extant specimens comprised 30 atlases of non-pathological adult *Homo*, *Pan*, *Gorilla* and *Pongo* sampling males and females, and 15 basicrania (humans and common chimpanzees only) (Supplementary Tables S1, S2).

### Virtual reconstruction of the atlas

The skull of StW 573 and the StW 679 atlas were scanned at the microfocus X-ray tomography facility of the Palaeosciences Centre at the University of the Witwatersrand, in Johannesburg (South Africa), at a spatial resolution of 88 µm and 19 µm, respectively (isotropic voxel size). All comparative specimens investigated in this study were imaged by X-ray tomography using various systems^58,59^ (Supplementary Tables S1, S2). Most of the comparative extant specimens have been downloaded from MorphoSource (https://www.morphosource.org/) and from the Digital Morphology Museum KUPRI (http://dmm.pri.kyoto-u.ac.jp/dmm/WebGallery/dicom/researcherTop.html; Supplementary Tables S1, S2). The cast of AL 333-83 has been rendered by using photogrammetry. Four of the extant human specimens have been imaged by using a Next Engine scanner.

The atlas of StW 573 was virtually extracted from the skull using Avizo v9.0 (Visualization Sciences Group Inc.) combining the watershed tool and manual corrections^60,61^. We reconstructed the missing portions of the left transverse process by mirroring the intact right transverse process (Fig. 2).

### Surface-based comparisons

We investigated morphology of the atlas by using a size-independent and landmark-free registration method based on smooth and invertible surface deformation^36,62^. As a pre-processing step, surfaces were automatically aligned in position, orientation and scale with respect to one surface randomly selected using the Iterative Closest Point (ICP) algorithm^63^. From this set of pre-aligned surfaces, an automatic non-rigid registration process was performed on the extant specimens only (i.e., *Homo*, *Pan*, *Gorilla*, and *Pongo*) via the deformation of a template using the software Deformetrica^36,62^ (available online at http://www.deformetrica.org/). A global mean shape and the deformation fields from the global mean shape to each extant specimen were computed from the set of aligned surfaces. Then, the global mean shape was subsequently deformed to StW 573, StW 679 and AL 333-83. Moreover, taxon mean shapes were generated for each extant group and deformed to StW 573.

Because StW 679 and AL 333-83 are incomplete, we performed a second analysis focusing on the left articular facets following the protocol published in Dumoncel *et al*.^35^ and Beaudet *et al*.^36^. Non-common regions of the atlases (i.e., regions not preserved in StW 679 and AL 333-83) were automatically eliminated from the sample so that all of the specimens contain only comparable information that can be used for the new registration process. This step has been automatically performed by using the deformation of the global mean shape to the extant and fossil specimens of the first analysis (see Fig. 1 in Beaudet *et al*.^36^). Subsequently, partial atlases were re-aligned using the ICP algorithm and the same reference as we used for the first analysis. In this manner, we computed a second analysis, including a second global mean shape and corresponding taxon mean shapes, from the set of partial atlases of using extant specimens only. The global mean shape and the taxon mean shapes were subsequently deformed to the partial atlases of StW 573, StW 679 and AL 333-83, providing deformation fields from the global mean shape to each fossil specimen.

Deformation fields integrating local orientation and the amplitude of deformations from the global mean shape to each specimen were statistically analysed by a principal component analysis (PCA) using the package ade4 for R^64^ (Fig. 3). The fossil specimens were subsequently projected into shape space. Thus, we computed two separate PCAs, i.e., a first one with the complete atlases (excluding StW 679 and AL 333-83, Fig. 3a) and a second one with the partial atlases (including the two partial fossil specimens and StW 573, Fig. 3b). Cumulative displacement variations are rendered and visualized using a pseudo-colour scale ranging from dark blue (lowest values) to red (highest values; Fig. 3). In addition to colour maps, we used vectors representing local maxima of the displacements. We computed a backtransform morphospace, i.e., representative shapes computed from the deformation fields are superimposed to the PCA to show how shape varies across the morphospace (see Olsen^65^ for an example based on 2D landmarks; Fig. 3). Colour scale represents variation from the global mean shape computed for the entire extant sample.

### Linear measurements

We used standard measurements reported in Gómez-Olivencia *et al*.^44^ and illustrated in Figure S2A for measuring dimensions of the atlas of StW 573 and those in the comparative extant sample. Because StW 679 and AL 333-83 are incomplete and could not be virtually reconstructed due to the absence of the anterior and posterior vertebral arches, we could not estimate on them any of the metric variables used in this study, except for the size of the left superior articular facet in StW 679. We used the product of the diameter in the major axis (1L/R) and the orthogonal diameter (2L/R) of each facet as an estimation of the area of the articular surface. Moreover, we divided the diameter in the major axis by the orthogonal diameter to assess a ratio between the length and breadth of each facet.

### Cross-sectional areas of the vertebral foramen and transverse foramina

We measured cross-sectional areas of the vertebral foramen (VFA) and the right (RFA) and left (LFA) transverse foramina (Supplementary Fig. S2B). In order to do this, we first defined the best-fit plane to the atlas using the module ‘Points to Fit’ in Avizo v9.0. This plane was moved to pass between superior and inferior articular facets when measuring the VFA, and through the most lateral points of the right and left transverse processes when measuring the RFA and LFA. A 2D section was extracted for both positions. Cross-sectional areas of the vertebral foramen and of the foramina were measured on the respective 2D sections by segmenting the canal and foramina using the module ‘Material statistics’ in Avizo v9.0. For standardizing measurements, we divided areas by the product of the length (i.e., from the most lateral tip of the right transverse process to the most lateral tip of the left transverse process) and width of the atlas (i.e., the most anterior point of the anterior arch to the most posterior point of the posterior arch). Only RFA could be measured in StW 679, while AL 333-83 preserves neither transverse process (Fig. 2).

### Cross-sectional area of the carotid canal

To avoid any potential distortions related to the presence of bony eminences surrounding the external opening or to torsion of the canal, we measured cross-sectional areas (CSA) of the left carotid canal at mid-distance between the external opening of the canal and the bending of the canal observed when entering the petrous bone (Supplementary Fig. S3). First, we placed landmarks on the aperture and defined a best-fit plane using the module ‘Points to Fit’ in Avizo v9.0 (Supplementary Fig. S3). This position was considered to represent the external opening of the canal (plane C in Supplementary Fig. S3). This plane was moved until reaching the ‘elbow’ of the canal in the petrous bone, after which the position was noted as representing the ‘elbow’ of the canal (plane A in Supplementary Fig. S3). Subsequently, the plane was moved and positioned equidistant between the two previously identified positions (i.e., the opening and the ‘elbow’) before virtually extracting the corresponding 2D plane and subjecting it to further measurements (plane B in Supplementary Fig. S3). Cross-sectional area of the carotid canal was measured by segmenting the canal and using the module ‘Material statistics’ in Avizo v9.0. As the portion of the carotid canal between the opening and the ‘elbow’ is partially filled with sediments in SK 847, we extracted a plane parallel to the plane passing through the foramen and that samples the best-preserved part of the bony canal. Accordingly, this measurement could not be directly compared to the rest of the sample, but it could be used as an approximation of the early *Homo* condition.

### Estimation of brain glucose utilization

We estimated brain glucose utilization (BGU) in StW 573 by using the equation provided by Boyer and Harrington^24^, i.e., ln(BGU) = 1.17*ln(ACA)−0.10, where ACA represents twice the sum of the respective averages of transverse foramen cross-sectional area and carotid canal cross-sectional area. Since the endocast of StW 573 is distorted^36^, we could not include the cranial capacity in the equation as recommended by the authors^24^. For extant comparative specimens, we used the mean, minimum and maximum values of CSA, RFA and LFA for computing the mean, minimum and maximum values of ACA (Table 4 and Supplementary Table S3). For fossil comparative specimens, we used cross-sectional area of the transverse foramen preserved in StW 679 as a proxy estimate for *Australopithecus* specimens. Because of the degree of variation in the measurement of the cross-sectional area of the transverse foramina of extant samples (e.g., up to 7% of differences between the right and left transverse foramina in *Homo*, Table 2), and because we could not account for inter-individual variation in transverse foramina in the *Australopithecus* sample, our computations of the ACA in the comparative *Australopithecus* specimens should be considered as initial provisional estimations. However, we tentatively estimated ranges of variations for fossil specimens by using the standard deviation of RFA and LFA of extant humans and extant chimpanzees and recomputing values for ACA and BGU (Table 4).

## Supplementary information

Supplementary Information.

## Data Availability

Permission to access and use 3D surfaces of the StW 573 and StW 679 atlases might be granted by submitting a request to the curator of the Evolutionary Studies Institute (B. Zipfel) via MorphoSource (Sterkfontein project, https://www.morphosource.org/Detail/ProjectDetail/Show/project_id/632).
