# Supplementary material for: The atlas of StW 573 and the late emergence of human-like head mobility and brain metabolism
Source: Sci Rep. 2020 Mar 16;10:4285. doi: 10.1038/s41598-020-60837-2 (PMC7075956; doi:10.1038/s41598-020-60837-2)
Supplement: Supplementary file 1 — Supplementary Information. [file 41598_2020_60837_MOESM1_ESM.pdf]

Supplementary Information for **The atlas of StW 573 and the late emergence of human-like head mobility and brain metabolism**

Authors

Amélie Beaudet[a,b,*], Ronald J. Clarke[c], Jason L. Heaton[c,d,e], Travis R. Pickering[c,d,f], Kristian J. Carlson[c,g], Robin H. Crompton[c,h], Tea Jashashvili[c,i,j], Laurent Bruxelles[a,k,l], Kudakwashe Jakata[c], Lunga Bam[m], Luc Van Hoorebeke[n], Kathleen Kuman[a], Dominic Stratford[a]

Author affiliations

[a]*School of Geography, Archaeology and Environmental Studies, University of the Witwatersrand, Private Bag 3, Johannesburg, WITS 2050, South Africa*

[b]*Department of Anatomy, University of Pretoria, PO Box 2034, Pretoria 0001, South Africa*

[c]*Evolutionary Studies Institute, University of the Witwatersrand, Private Bag 3, Johannesburg, WITS 2050, South Africa*

[d]*Department of Biology, Birmingham-Southern College, 900 Arkadelphia Road, Birmingham, AL 35254, United States*

[e]*Plio-Pleistocene Palaeontology Section, Department of Vertebrates, Ditsong National Museum of Natural History (Transvaal Museum), 432 Paul Kruger Street, Pretoria Central, Pretoria, South Africa*

[f]*Department of Anthropology, University of Wisconsin, Madison, WI 53706, United States*

[g]*Department of Integrative Anatomical Sciences, Keck School of Medicine, University of Southern California, 1975 Zonal Avenue, Los Angeles, CA 90033, United States*

[h]*Department of Musculoskeletal Biology, Institute of Ageing and Chronic Disease, University of Liverpool, William Henry Duncan Building, W Derby Street, Liverpool, L7 8TX, United Kingdom*

[i]*Molecular Imaging Center, Department of Radiology, Keck School of Medicine, University of Southern California, 1975 Zonal Avenue, Los Angeles, CA 90033, United States*

[j]*Department of Geology and Paleontology, Georgian National Museum, 3/10 Shota Rustaveli Ave, T'bilisi 0105, Georgia*

[k]*French National Institute for Preventive Archaeological Researches (INRAP), 561 rue Etienne Lenoir, 30900 Nîmes, France*

[l]*French Institute of South Africa (IFAS), USR 3336 CNRS, 62 Juta Street, Braamfontein, Johannesburg 2001, South Africa*

[m]*South African Nuclear Energy Corporation SOC Ltd. (Necsa), Elias Motsoaledi Street Ext. (Church Street West), R104 Pelindaba, North West Province, South Africa*
[n]*UGCT Department of Physics and Astronomy, Ghent University, Proeftuinstraat 86/N12, B-9000 Gent, Belgium*

This PDF file includes:

Figures S1 to S3

Tables S1 to S3

References SI reference citation

Figure S1. Surface-based comparison of fossil partial atlases. Topographical distribution of deformation-based shape comparisons of the partial atlases StW 679 and AL 333-83 to StW 573. Cumulative displacement variations (**a**) from StW 679 (grey surface) to StW 573 and (**b**) from AL 333-83 (grey surface) to StW 573 are rendered by a pseudo-colour scale ranging from dark blue (lowest values) to red (highest values) at the fossil individual surfaces. Vectors represent both the magnitude and orientation of deformations. The maximum value of the colour bar is considered to be the most appropriate compromise representation of both global and local deformations. Atlases are shown in superior views.

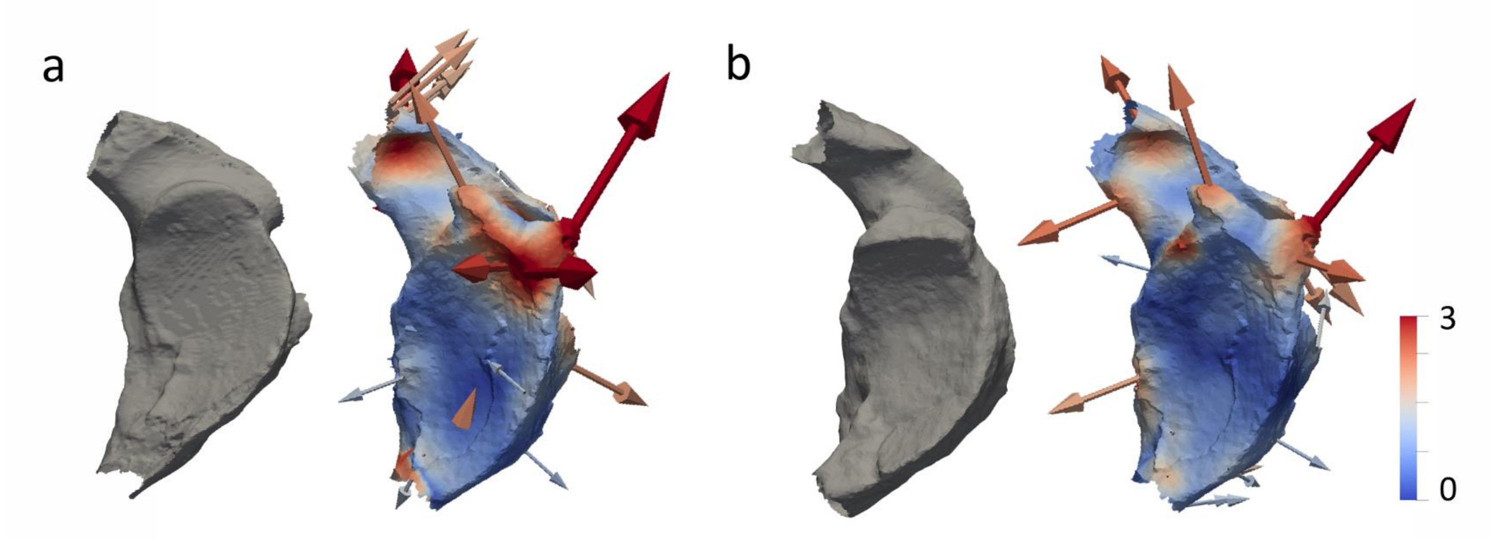

Figure S2. Linear measurements and cross-sectional areas. (**a**) Linear measurements assessed in the atlas of StW 573 and of comparative specimens in superior view. (**b)** Cross-sectional areas (transverse foramina in green and vertebral foramen in red) assessed in the atlas of StW 573 and of comparative specimens in inferior views.

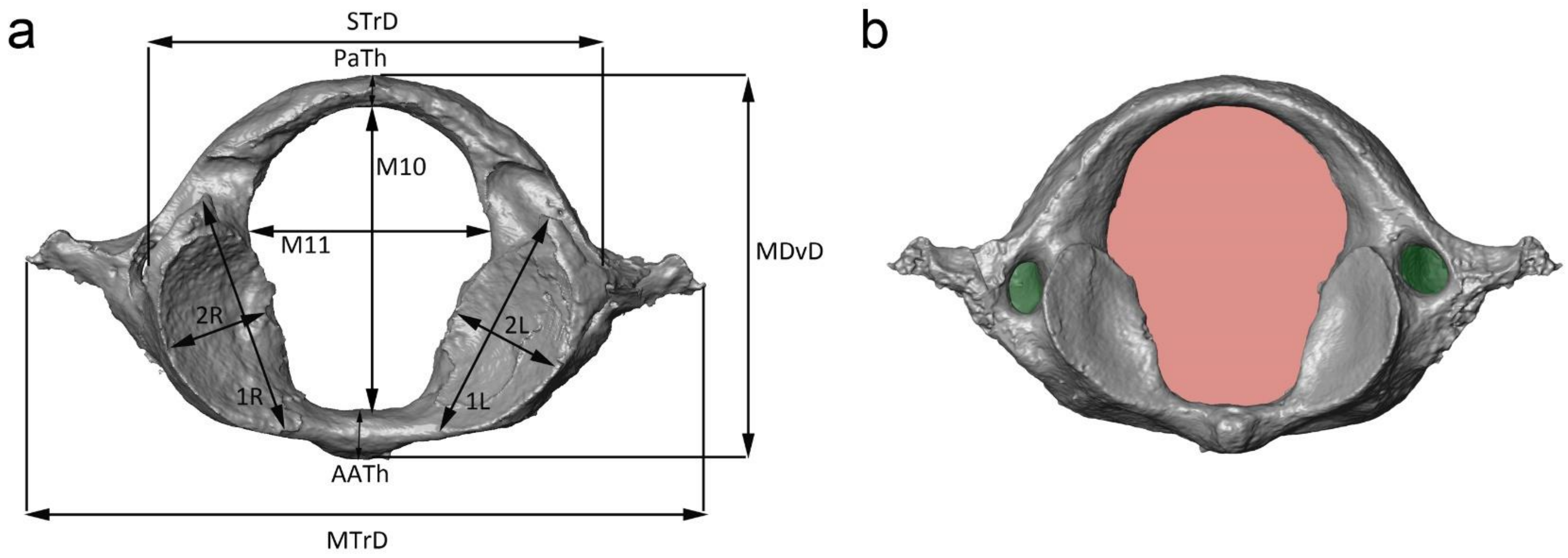

Figure S3. Protocol for assessing cross-sectional area (CSA) of the carotid canal applied to an extant *Homo* specimen. Landmarks are placed on the aperture (left, basicranium in grey) and defined using a best-fit plane that was translated until reaching the ‘elbow’ of the carotid canal (bottom right, 3D virtual reconstruction of the carotid canal in blue). Three sections were extracted from the temporal bone corresponding to the external opening of the canal (plane a), the ‘elbow’ of the canal observed when entering the petrous bone (plane c) and the plane at mid-distance between plane a and plane c (i.e., plane b). Blue areas represent the carotid canal.

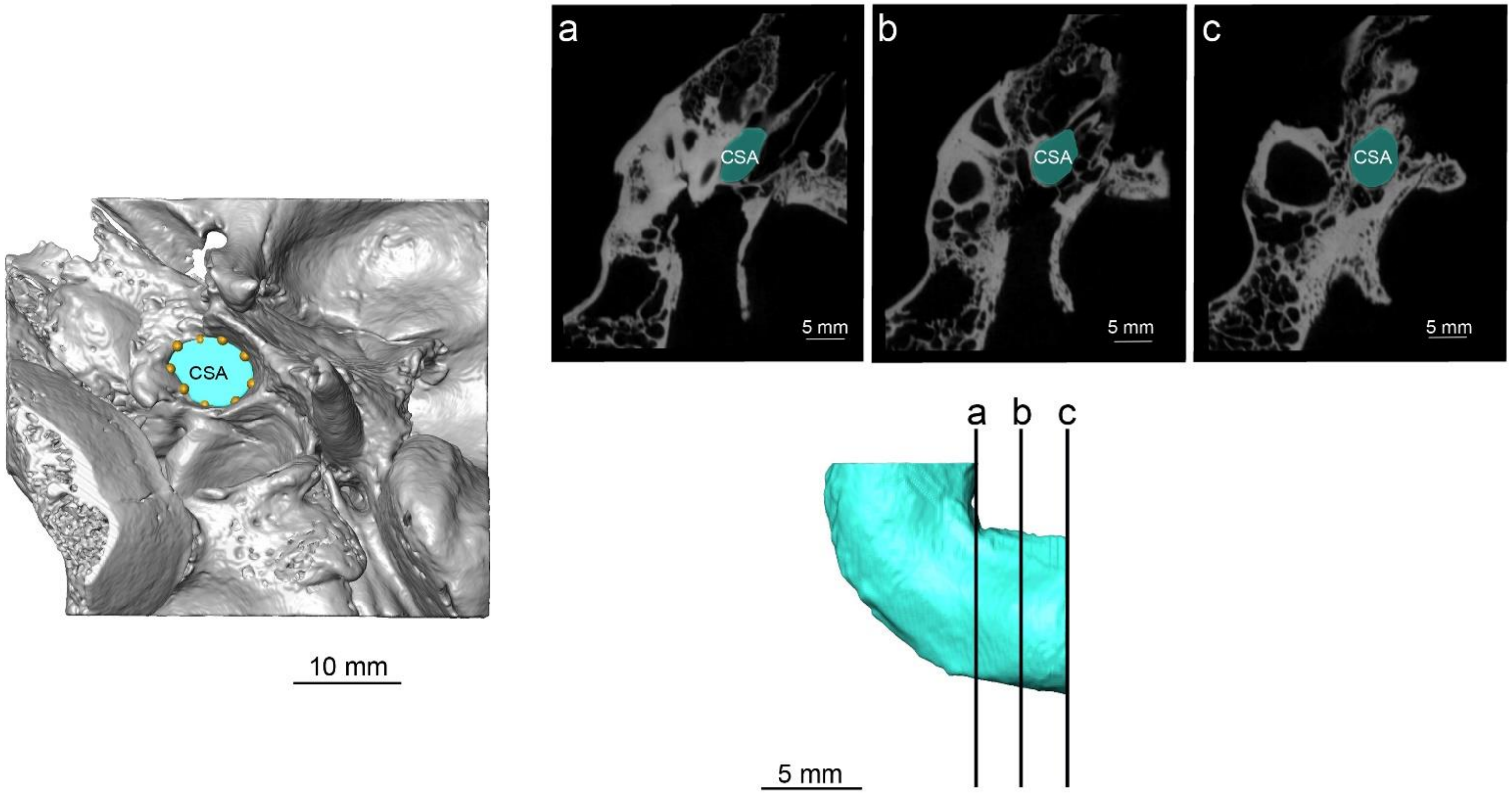

Table S1. Comparative sample used for assessing variation in osteological dimensions and morphology of the atlas.

specimen/ sample	site	taxonomic attribution	sex	references	stored at	imaging facility	voxel size (mm)	GM analyses	linear/surface measures
fossil specimens									
AL 333-83	Hadar Formation	*Australopithecus afarensis*	?	1	ESI	photogram-metry	-	*n* = 1	-
extant specimens									
Homo (*n* = 9)		*H. sapiens*	F (*n* = 5)<br>M (*n* = 3)<br>? (*n* = 1)	2	PBC<br>DU	Necsa<br>SMiF	0.039-0.750	*n* = 9	*n* = 9
Pan (*n* = 12)		*P. troglodytes*<br>*P. paniscus*	F (*n* = 7)<br>M (*n* = 3)<br>? (*n* = 2)		RMCA<br>PRI<br>AMNH<br>YPBM	UGCT<br>PRI<br>DID<br>Y-TRIC	0.070-0.430	*n* = 12	*n* = 12
Gorilla (*n* = 6)		*G. gorilla*,<br>*G. beringei*	F (*n* = 2)<br>M (*n* = 3)<br>? (*n* = 1)		RMCA<br>PRI<br>YPBM	UGCT<br>PRI<br>Y-TRIC	0.070-0.660	*n* = 6	*n* = 6
Pongo (*n* = 3)		*P. abelii*<br>*P. pygmaeus*	F (*n* = 1)<br>M (*n* = 2)		PRI	PRI	0.500-0.625	*n* = 1	*n* = 3

AMNH: American Museum of Natural History; PBC: Pretoria Bone Collection, University of Pretoria, South Africa; DID: Diagnostic Imaging Department of the NC State Veterinary Hospital, USA; DU: Department of Evolutionary Anthropology, Duke University, USA; ESI: Evolutionary Studies Institute, South Africa; Necsa: South African Nuclear Energy Corporation, South Africa[26]; PRI: Primate Research Institute, Kyoto University, Japan; RMCA: Royal Museum for Central Africa, Belgium; SMiF: Shared Materials Instrumentation Facility, Duke University, USA; UGCT: Centre for X-ray Tomography of Ghent University, Belgium[27]; YPBM: Peabody Museum of Natural History, Yale University, USA; Y-TRIC: Yale Transitional Research Imaging Center, USA. F: female; M: male: ?: no information available.

Table S2. Comparative sample used for assessing variation in the cross-sectional area of the internal carotid canal.

specimen/ sample	site	taxonomic attribution	sex	references	stored at	imaging facility	voxel size (mm)
fossil specimens							
Sts 5	Sterkfontein Member 4	*Australopithecus africanus*	F	3-7	DNMNH	Pal. Centre	0.075
Sts 19	Sterkfontein Member 4	*Australopithecus africanus*	F	4, 8-9	DNMNH	Necsa	0.044
Sts 25	Sterkfontein Member 4	*Australopithecus* sp.	?	10-12	ESI	Pal. Centre	0.065
StW 53	Sterkfontein Member 4*	*Australopithecus africanus*	M	6, 13-17	ESI[b]	Pal. Centre	0.025
StW 98	Sterkfontein Member 4	*Australopithecus* sp.	?	18	ESI	Pal. Centre	0.033
StW 329	Sterkfontein Member 4	*Australopithecus africanus*	?	18	ESI	Pal. Centre	0.033
StW 498	Sterkfontein Member 4	*Australopithecus prometheus*	?	6, 18	ESI	Pal. Centre	0.028
MLD 37/38	Makapansgat Member 4	*Australopithecus africanus*	F	19-20	ESI	Pal. Centre	0.080
SK 47	Swartkrans Member 1	*Paranthropus robustus*	F	21-22	DNMNH	Pal. Centre	0.071
SK 847	Swartkrans Member 1	*Homo erectus/Homo ergaster*	?	23-25	DNMNH	Max Planck	0.063
extant specimens							
Homo (*n* = 10)		*H. sapiens*	F (*n* = 5) M (*n* = 5)	2	PBC	Necsa	0.099-0.114
Pan (*n* = 5)		*P. troglodytes*	F (*n* = 1) M (*n* = 4)		RMCA	UGCT	0.075-0.085

DNMNH: Ditsong National Museum of Natural History, South Africa; ESI: Evolutionary Studies Institute, South Africa; Necsa: South African Nuclear Energy Corporation, South Africa[26]; Max Planck: Max Planck Society, Department of Human Evolution, Virtual Reality Laboratory, Germany; Pal. Centre: Palaeosciences Centre, University of the Witwatersrand, South Africa; PBC: Pretoria Bone Collection, University of Pretoria, South Africa; RMCA: Royal Museum for Central Africa, Belgium; UGCT: Centre for X-ray Tomography of Ghent University, Belgium[27].
*16, 28

Table S3. Estimation of brain glucose utilization (BGU) in StW 573 and comparative material.

specimen/sample	ACA	BGU
StW 573	57.3	103.2
Australopithecus		
Sts 5	51.2	90.5
Sts 19	56.2	100.9
Sts 25	54.4	97.1
StW 53	70.6	131.7
StW 98	55.4	99.2
StW 329	48.8	85.5
StW 498	58.0	104.7
MLD 37/38	46.8	81.4
mean	55.2	98.9
range	46.8-70.6	81.4-131.7
extant *Homo*		
mean	131.8	273.5
range	96.4-206.3	189.6-461.9
extant *Pan*		
mean	68.5	127.2
range	33.9-102.1	55.8-202.8

ACA: total arterial cross-sectional area (mm^2).
For each *Australopithecus* individual besides StW 573, we use the estimate of cross-sectional area of the transverse foramen of StW 679 for a proxy value and combine these with cross-sectional areas of carotid canal measures provided in Table 3.
For extant *Homo* and *Pan*, we use the taxon-specific estimates provided in Tables 2-3.

References

1. Lovejoy, C.O., Johanson, D.C. & Coppens, Y. Elements of the axial skeleton recovered from the Hadar formation: 1974-1977 collections. *Am. J. Phys. Anthropol.* **57**, 631–635 (1982).
2. L'Abbé, E.N., Loots, M. & Meiring, J. H. The Pretoria Bone Collection: a modern South African skeletal sample. *Homo* **56**, 197–205 (2005).
3. Broom, R. Discovery of a new skull of the South African ape-man, *Plesianthropus*. *Nature* **159**, 672 (1947).
4. Broom, R. & Robinson, J.T. Further evidence of the structure of the Sterkfontein ape-man *Plesianthropus*. *Transv. Mus. Mem.* **4**, 7–83 (1950).
5. Broom, R., Robinson, J.T. & Schepers, G.W.H. Sterkfontein ape-man, *Plesianthropus*. *Transv. Mus. Mem.* **4**, 1–117 (1950).
6. Clarke, R.J. Latest information on Sterkfontein's *Australopithecus* skeleton and a new look at *Australopithecus*. *S. Afr. J. Sci.* **104**, 443–449 (2008).
7. Grine, F.E., Weber, G.W., Plavcan, J.M. & Benazzi, S. Sex at Sterkfontein: 'Mrs. Ples' is still an adult female. *J. Hum. Evol.* **62**, 593–604 (2012).
8. Tobias, P.V. & Hughes, A.R. 1969. The new Witwatersrand University excavation at Sterkfontein. *S. Afr. Archaeol. Bull.* **24**, 158–169.
9. Thackeray, J.F. & Gommery, D. Spatial distribution of australopithecine specimens discovered at Sterkfontein between 1947 and 1949. *Annls. Transv. Mus.* **39**, 70–72 (2002).
10. Brain, C.K. *The Hunters of the Hunted? An introduction to African Cave Taphonomy* (University of Chicago Press, 1981).
11. Wolpoff, M.H. & Lee, S.H. Variation in the habiline crania – Must it be taxonomic? *Hum. Evol.* **21**, 71–84 (2006).
12. Grine, F.E. The alpha taxonomy of *Australopithecus africanus*. in *The Paleobiology of* Australopithecus (eds. Reed, K., Fleagle, J., & Leakey, R.) 74–104 (Springer, 2013).
13. Hughes, A.R. & Tobias, P.V. A fossil skull probably of the genus *Homo* from Sterkfontein, Transvaal. *Nature* **265**, 310–312 (1977).
14. Clarke, R.J. On some new interpretations of Sterkfontein stratigraphy. *S. Afr. J. Sci.* **90**, 211–214 (1994).
15. Clarke, R.J. *Homo habilis*: The inside story in *Proceedings of the II Meeting of African Prehistory* (eds. Sahnouni, M., Semaw, S. & Garaizar, J.R.) 25–51 (Consorcio CENIEH, 2017).

16. Kuman, K. & Clarke, R.J. Stratigraphy, artefact industries and hominid associations for Sterkfontein, Member 5. *J. Hum. Evol.* **38**, 827–847 (2000).
17. Thackeray, J.F. *et al*. Comparison of Sts 5 ('Mrs Ples') and Stw 53 ('Early *Homo*') from Sterkfontein, South Africa. *S. Afr. J. Sci*. **96**, 21 (2000).
18. Lockwood, C.A. & Tobias, P.V. Morphology and affinities of new hominid cranial remains from Member 4 of the Sterkfontein Formation, Gauteng Province, South Africa. *J. Hum. Evol.* **42**, 389–450 (2002).
19. Dart, R.A. The first *Australopithecus* cranium from the pink breccia at Makapansgat. *Am. J. Phys. Anthropol.* **17**, 77–82 (1959).
20. Dart, R.A. The Makapansgat pink breccia Australopithecine skull. *Am. J. Phys. Anthropol.* **20**, 119–126 (1962).
21. Broom, R. & Robinson, J.T. “Swartkrans Ape-Men. *Paranthropus crassidens*.” (Transvaal Museum, 1952).
22. Dean, M.C. & Wood, B.A. Basicranial anatomy of Plio-Pleistocene hominids from East and South Africa. *Am. J. Phys. Anthropol.* **59**, 157–174 (1982).
23. Clarke, R.J. & Howell, F.C. Affinities of the Swartkrans 847 hominid cranium. *Am. J. Phys. Anthropol.* **37**, 319–325 (1972).
24. Clarke, R.J. The cranium of the Swartkrans hominid, SK 847 and its relevance to human origins *Ph.D. Dissertation* (University of the Witwatersrand, 1977).
25. Clarke, R.J. The history of research in human evolution in Africa and what lessons have been learned in *Human Origin Sites and the World Heritage Convention in Africa* (ed. Sanz, N.) 44–67 (United Nations Educational, Scientific and Cultural Organization, 2012).
26. Hoffman, J.W. & de Beer, F.C. Characteristics of the micro-focus X-ray tomography facility (MIXRAD) at Necsa in South Africa in *18th World Conference of Nondestructive Testing, Durban* (2012). http://www.ndt.net/article/wcndt2012/papers/37_wcndtfinal00037.pdf.
27. Masschaele, B. *et al*. HECTOR: A 240kV micro-CT setup optimized for research. *J. Phys. Conf. Ser.* **463**, 012012 (2013).
28. Clarke, R.J. *Australopithecus* from Sterkfontein Caves, South Africa in *The Paleobiology of* Australopithecus (eds. Reed, K., Fleagle, J., & Leakey, R.) 105–123 (Springer, 2013).
